# Formation of nuclear CPSF6/CPSF5 biomolecular condensates upon HIV-1 entry into the nucleus is important for productive infection

**DOI:** 10.1038/s41598-023-37364-x

**Published:** 2023-07-06

**Authors:** Charlotte Luchsinger, KyeongEun Lee, Gonzalo A. Mardones, Vineet N. KewalRamani, Felipe Diaz-Griffero

**Affiliations:** 1 Department of Microbiology and Immunology, Albert Einstein College of Medicine, 1301 Morris Park – Price Center 501, Bronx, NY 10461 USA; 2 Basic Research Laboratory, Center for Cancer Research, National Cancer Institute at Frederick, Frederick, MD 21702 USA; 3 Facultad de Medicina Y Ciencia, Universidad San Sebastian, Arturo Prat 154, Valdivia, Chile

**Keywords:** Cell biology, Microbiology, Molecular biology

## Abstract

The early events of HIV-1 infection involve the transport of the viral core into the nucleus. This event triggers the translocation of CPSF6 from paraspeckles into nuclear speckles forming puncta-like structures. Our investigations revealed that neither HIV-1 integration nor reverse transcription is required for the formation of puncta-like structures. Moreover, HIV-1 viruses without viral genome are competent for the induction of CPSF6 puncta-like structures. In agreement with the notion that HIV-1 induced CPSF6 puncta-like structures are biomolecular condensates, we showed that osmotic stress and 1,6-hexanediol induced the disassembly of CPSF6 condensates. Interestingly, replacing the osmotic stress by isotonic media re-assemble CPSF6 condensates in the cytoplasm of the cell. To test whether CPSF6 condensates were important for infection we utilized hypertonic stress, which prevents formation of CPSF6 condensates, during infection. Remarkably, preventing the formation of CPSF6 condensates inhibits the infection of wild type HIV-1 but not of HIV-1 viruses bearing the capsid changes N74D and A77V, which do not form CPSF6 condensates during infection^1,2^. We also investigated whether the functional partners of CPSF6 are recruited to the condensates upon infection. Our experiments revealed that CPSF5, but not CPSF7, co-localized with CPSF6 upon HIV-1 infection. We found condensates containing CPSF6/CPSF5 in human T cells and human primary macrophages upon HIV-1 infection. Additionally, we observed that the integration cofactor LEDGF/p75 changes distribution upon HIV-1 infection and surrounds the CPSF6/CPSF5 condensates. Overall, our work demonstrated that CPSF6 and CPSF5 are forming biomolecular condensates that are important for infection of wild type HIV-1 viruses.

## Introduction

Infection of human cells by HIV-1 requires fusion of the viral membrane with the cellular membrane, which delivers the viral core into the cytoplasm of the cell. The viral core, composed of 1500 monomers of the capsid protein (p24), houses the viral RNA genome. This viral core travels to the nucleus, where it has been suggested that the RNA is converted into viral DNA by reverse transcription^2–4^. While reverse transcription occurs, the viral core undergoes uncoating, which is the dissociation of monomeric capsids proteins from the core resulting in the opening of the structure that houses the viral genome. Concomitant with these early replication events, the nuclear cleavage and polyadenylation specific factor 6 (CPSF6) is recruited from nuclear paraspeckles to nuclear speckles (NS)^1,2,5^. NS are dynamic borderless structures that are distinct entities in the nucleus.

The early recruitment of CPSF6 to NS induced by HIV-1 infection requires an intact capsid protein. Viruses with capsid mutations N74D or N57S fail to induce the translocation of CPSF6 to NS^1,2^. Translocation of CPSF6 to NS induced by HIV-1 infection changes the immunofluorescence microscopy pattern of CPSF6 from diffuse nuclear staining to large CPSF6 condensates or puncta-like structures that are easily recognizable^1,2^. Although the translocation of CPSF6 to NS is widely accepted, the role of this translocation in HIV-1 replication, if any, is not understood.

NS, which are important for the expression of highly active genes, comprise protein and RNA that coordinate transcription and splicing^6–8^. NS are also known as SC35-rich domains^9^ as they are easily visualized by antibodies directed against the spliceosome assembly factor SC35^10^. One possibility is that the recruitment of CPSF6 to nuclear speckles plays an important role in replication. The addition of the small pharmacological molecules PF74, GS-CA-1, or lenacapavir during infection inhibits translocation of CPSF6 to NS^11^, and PF74 disassembles CPSF6 condensates preformed by HIV-1 infection. In contrast, neither GS-CA-1 nor lenacapavir triggers CPSF6 disassembly, indicating that they act by a different mechanism than PF74^11^. Contrary to protein aggregates in the cell, which are poorly dynamic, the ability of small molecules to destabilize the CPSF6 condensates suggests that they are dynamic structures.

The contribution of HIV-1-induced CPSF6 condensates to infection is not understood. Although CPSF6 interacts with the viral capsid, the lack of CPSF6 has no effect on HIV-1 infection of stimulated primary human T cells ^11^. However, the absence of CPSF6 does affect integration site selection for HIV-1, suggesting that, like LEDGF/p75, CPSF6 is involved in the selection of integration sites in the host genome^12,13^. Furthermore, infection of CPSF6 knockout cells by HIV-1 results in an integration pattern similar to the integration pattern observed for HIV-1-N74D viruses^12,13^; these results imply that in the absence of CPSF6 expression, HIV-1 wild type virus behaves like HIV-1-N74D viruses. Therefore, knocking out CPSF6 expression may not be the best approach to understand its contribution to wild type HIV-1 infection. Clearly, it has been shown that the absence of CPSF6 hinders the reactivation of latent proviruses^14^.

The translocation of CPSF6 to NS induced by HIV-1 infection requires an intact capsid since viruses containing capsid mutations such as N74D and A77V fail to induce condensates containing CPSF6^1,2^. We initially investigated whether reverse transcription and/or integration are required for the formation of CPSF6 condensates. We found that neither reverse transcription nor integration were required for the formation of CPSF6 condensates. Similarly, the HIV-1 genome was not required for the formation of CPSF6 condensates. Because CPSF6 forms condensate-like structures in the nucleus upon HIV-1 infection, we determined whether these puncta-like structures were biomolecular condensates. To this end, we tested whether osmotic stress and the drug 1,6-hexanediol affected the nature and stability of these condensates. We found that hypertonic stress resulted in the disassembly of CPSF6 condensates, and reestablishing isotonic conditions resulted in the reassembly of CPSF6 condensates in the nucleus. By contrast, 1,6-hexanediol resulted in the disassembly of CPSF6 condensates, and the removal of the drug allowed reassembly of CPSF6 condensates in the cytoplasm. HIV-1-infected human cells transferred from an hypotonic to an isotonic medium reassembled CPSF6 condensates in the cytoplasm. Thus, unlike protein aggregates, CPSF6 condensates are dynamic structures that behave like biomolecular condensates. The HIV-1 inhibitor GS-CA-1 prevented the disassembly of nuclear CPSF6 condensates triggered by hypotonic stress; thus, GS-CA-1 stabilizes CPSF6 condensates under these conditions. To determine whether CPSF6 condensates play a role in infection, we challenged human cells with HIV-1 in a hypertonic medium that prevents the assembly of CPSF6 condensates during infection. Remarkably, the hypertonic medium dramatically inhibited wild-type HIV-1 infection, but poorly affected viruses bearing the capsid changes N74D and A77V, which are viruses that do not induce the formation of CPSF6 condensates^1,2^. Thus, the CPSF6 condensates induced by wild-type HIV-1 are important for infection. To further characterize the protein content of these structures, colocalization experiments identified the protein CPSF5 but not CPSF7 as part of the condensate. Our studies revealed that the accumulation of CPSF6 biomolecular condensates in NS is important for wild-type HIV-1 infection.

## Results

### CPSF6 is recruited to NS in several human cell lines after HIV-1 infection

**Figure 1:**
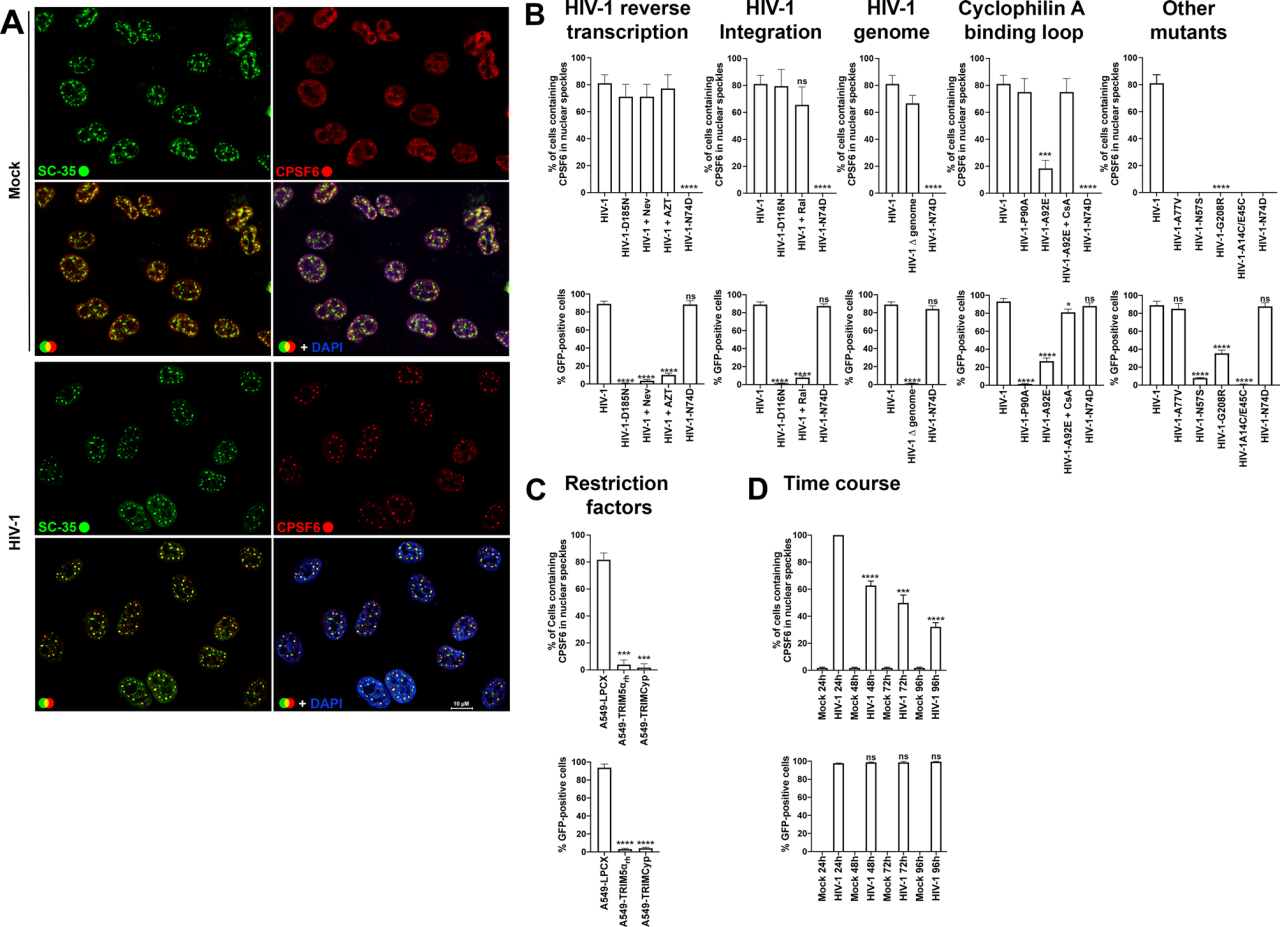
**Viral mutations, inhibitors, and restriction factors affect the recruitment of CPSF6 to NS.** (**A**) To induce the formation of CPSF6 condensates, A549 cells were challenged with HIV-1-Luc viruses at an MOI of ~ 2 for 24 h. Subsequently, cells were fixed, permeabilized, and immunolabeled using specific antibodies directed against SC35 (green) and CPSF6 (red). Secondary antibodies were Cy5-conjugated donkey anti-mouse IgG and Alexa-594-conjugated donkey anti-rabbit IgG, respectively. For Nuclei were stained with DAPI (blue). Cells were examined by fluorescence microscopy. Merging the red and green channels generated the lower left image; yellow indicates the overlapping location of the red and green channels. Merging all channels generated the lower right image. (**B**) To evaluate the effect of viral mutations and inhibitors in the formation of CPSF6 condensates, we infected A549 cells with wild-type or the indicated mutant HIV-1-GFP viruses for 24 h using an MOI of ~ 2 when possible or normalized by p24. A549 cells were also infected with wild-type HIV-1-GFP with reverse transcription inhibitors 10 μM nevirapine (Nev) and 10 μM zidovudine (AZT), the integration inhibitor 10 μM raltegravir (Ral), or 5 μM cyclosporin A (CsA), as indicated. Cells were fixed, permeabilized, and immunolabeled with rabbit anti-CPSF6 and mouse anti-SC35 antibodies. The secondary antibodies were Alexa-594-conjugated donkey anti-rabbit IgG and Cy5-conjugated donkey anti-mouse IgG. The percentage of cells containing CPSF6 in nuclear speckles was determined by visual inspection of 200 cells per sample in three independent experiments. In parallel infectivity was assayed by measuring the % of GFP-positive cells for in three independent experiments (lower panels). (**C**) To evaluate the effect of restriction factors in the recruitment of CPSF6 to NS, we infected A549 cells stably expressing the restriction factors TRIM5α_rh_ or TRIMCyp with HIV-1-GPF at an MOI of ~ 2 for 24 h. The control experiment used the empty vector LPCX. Cells were fixed, permeabilized, and immunolabeled, as in (**B**). The percentage of cells containing CPSF6 in NS was determined as in (**B**). Similarly, infectivity was assayed by measuring the percentage of GFP-positive cells (lower panel). (**D**) To determine the stability of CPSF6 condensates, we infected A549 cells with HIV-1-GFP at an MOI of ~ 2 for 24-, 48-, 72-, or 96-h as indicated. Uninfected cells were used as control (mock). At the indicated times, cells were fixed, permeabilized, and immunolabeled, as in (**B**). The percentage of cells containing CPSF6 condensates in the nucleus (N) or in the cytosol (C) was determined by visual inspection of 80 cells per sample and normalized to the total amount of cells in three independent experiments (n = 240 cells). Data represent the mean ± standard deviation. **p ≤ 0.05, **p ≤ 0.0, ***p ≤ 0.001, ****p ≤ 0.0001; ns, not statistically significant; unpaired two-tailed t test.

We and others have previously demonstrated that HIV-1 infection induces the recruitment of CPSF6 to NS^1,2,5^. CPSF6 in uninfected human cells shows semi-diffuse nuclear labeling, whereas HIV-1 infection triggers the formation of nuclear condensates or puncta structures that contain CPSF6, which colocalize with the NS marker SC35 (Fig. 1A), as previously shown^1,2,5^. These condensates are easily visualized in cells by fluorescence microscopy, thereby providing a simple way to quantify cells containing these structures. To further explore the role of these condensates in infection, we used immunofluorescence microscopy to screen several human cell lines infected with HIV-1-GFP at a multiplicity of infection (MOI) 1–2 (infecting > 90% of HT1080 cells) for CPSF6 condensates. After infecting human cells THP-1 (monocytes), U937 (monocytes), HT1080 (epithelial), HeLa (epithelial), and A549 (epithelial) cells, we found that 80%–85% of A549 cells showed CPSF6 condensates in NS, defined as SC35 positive compartments. For HeLa and HT1080 cells, 30–40% of the cells contained CPSF6 condensates. In contrast, less than 1% of phorbol 12-myristate 13-acetate (PMA)-differentiated THP-1 and U937 cells had CPSF6 condensates. These results indicated that A549 cells provide a reliable model for studying the formation and function of CPSF6 condensates in NS after HIV-1 infection.

As a proof of principle, we infected A549 cells by an HIV-1 virus expressing luciferase as a reporter of infection (HIV-1-Luc) at an MOI =  ~ 1–2 and showed that infection triggers the translocation of CPSF6 to NS (Fig. 1A). On the contrary in mock infected cells CPSF6 labeling remained semi-diffuse nuclear (Fig. 1A).

### Viral mutations, inhibitors, and restriction factors affect the recruitment of CPSF6 to NS

We studied the role of HIV-1 determinants in the formation of CPSF6 condensates in NS using viral mutations and inhibitors. To this end, we simultaneously measure formation of CPSF6 condensates and infectivity. First, we found that the HIV-1 reverse transcriptase mutant D185N produced the same percentage of cells containing CPSF6 condensates when compared to the wild-type virus (Fig. 1B). HIV-1-D185N was normalized to p24 in the HIV-1-GFP wild-type virus by enzyme-linked immunosorbent assays (ELISA) and western blots. Similarly, reverse transcription inhibitors nevirapine (Nev) and zidovudine (AZT) did not affect the formation of CPSF6 condensates (Fig. 1B). Next, we determined that inhibition of viral integration by infection with HIV-1-D116N, which is a virus that has the integrase mutation D116N^15^, did not alter the formation of CPSF6 condensates in NS. HIV-1-D116N was normalized to p24 in the HIV-1-GFP wild-type virus by ELISA and/or western blots. Similarly, the integrase inhibitor raltegravir (Ral) did not affect the formation of CPSF6 condensates in NS. Overall, these results demonstrated that neither integration nor reverse transcription is required for the recruitment of CPSF6 to NS.

Because inhibition of reverse transcription did not affect the formation of CPSF6 condensates, we determined whether infection by viruses without a genome triggered the recruitment of CPSF6 to NS. Infection of A549 cells by an HIV-1 virus produced without a genome had no effect on the formation of CPSF6 condensates in NS (Fig. 1B). The HIV-1 virus without a genome was normalized to p24 in the HIV-1-GFP wild-type virus by ELISA and western blots. These results indicated that the viral RNA is unlikely to be involved in the formation of CPSF6 condensates.

To determine whether the cyclophilin A binding loop of the HIV-1 capsid contained determinants for the formation of CPSF6 condensates, we infected cells with an HIV-1 virus with the capsid change P90A, a mutation that prevents the binding of cyclophilin A to the capsid, which had no effect on the formation of CPSF6 condensates when compared to the wild-type HIV-1 (Fig. 1B). Interestingly, an HIV-1 virus with the cyclosporin A (CsA)-dependent capsid mutant A92E showed decreased formation of CPSF6 condensates, which was rescued by the addition of CsA (Fig. 1B), and the increase in HIV-1-A92E infection with CsA was proportional to the formation of nuclear CPSF6 condensates. HIV-1 mutant viruses were normalized to p24 in the HIV-1-GFP wild-type virus by ELISA and/or western blots.

As shown previously, the HIV-1 virus with the capsid change N74D did not induce the recruitment of CPSF6 to NS^1,2^. Similarly, infection by viruses with capsid mutations A77V, N57S, G208R, or A14C/E45C did not trigger the formation of CPSF6 condensates in NS compared to wild type (Fig. 1B). HIV-1 mutant viruses were normalized to p24 in the HIV-1-GFP wild-type virus by ELISA and/or western blots.

We previously demonstrated that restriction factors rhesus TRIM5α (TRIM5α_rh_) and owl monkey TRIMCyp prevent the entry of viral capsid into the nucleus^2^; therefore, we determined whether cells expressing these factors showed differences in the HIV-1-induced recruitment of CPSF6 to NS. Consistent with the requirement for the nuclear capsid in the recruitment of CPSF6 to NS^2^, expression of the restriction factors TRIM5α_rh_ and TRIMCyp prevented the formation of CPSF6 condensates in NS (Fig. 1C).

### Presence of CPSF6 condensates overtime

The formation of CPSF6 condensates is likely to be important for viral infection; therefore, we sought to determine whether the CPSF6 condensates are transient or stable structures since some viruses require the assembly and disassembly of condensates for the completion of their viral life cycle^16,17^. To this end, we challenged human A549 cells with HIV-1-GFP at an MOI ~ 2, which resulted in infection of > 90% of the cells at 24 h post-challenge as measured by flow cytometry. At 24-, 48-, 72-, and 96-h post-infection, we counted the number of cells containing CPSF6 condensates and normalized the % of cells containing condensates to the total number of cells. Interestingly, we found the highest percentage at 24 h post-infection (Fig. 1D). The percentage of cells containing CPSF6 condensates declined at 48-, 72-, and 96-h post-infection, suggesting that the formation of these structures was transient. As a control, we determined infection at every time point by measuring the percentage of GFP-positive cells (Fig. 1D, lower panel). Similar results were observed using the HIV-1-Luc virus (HIV-1_NL4-3_∆env-Luc) (data not shown). These experiments suggested that the CPSF6 condensates formed upon infection that serve an unknown function are subsequently disassembled.

### Osmotic stress affects the stability of CPSF6 condensates

**Figure 2:**
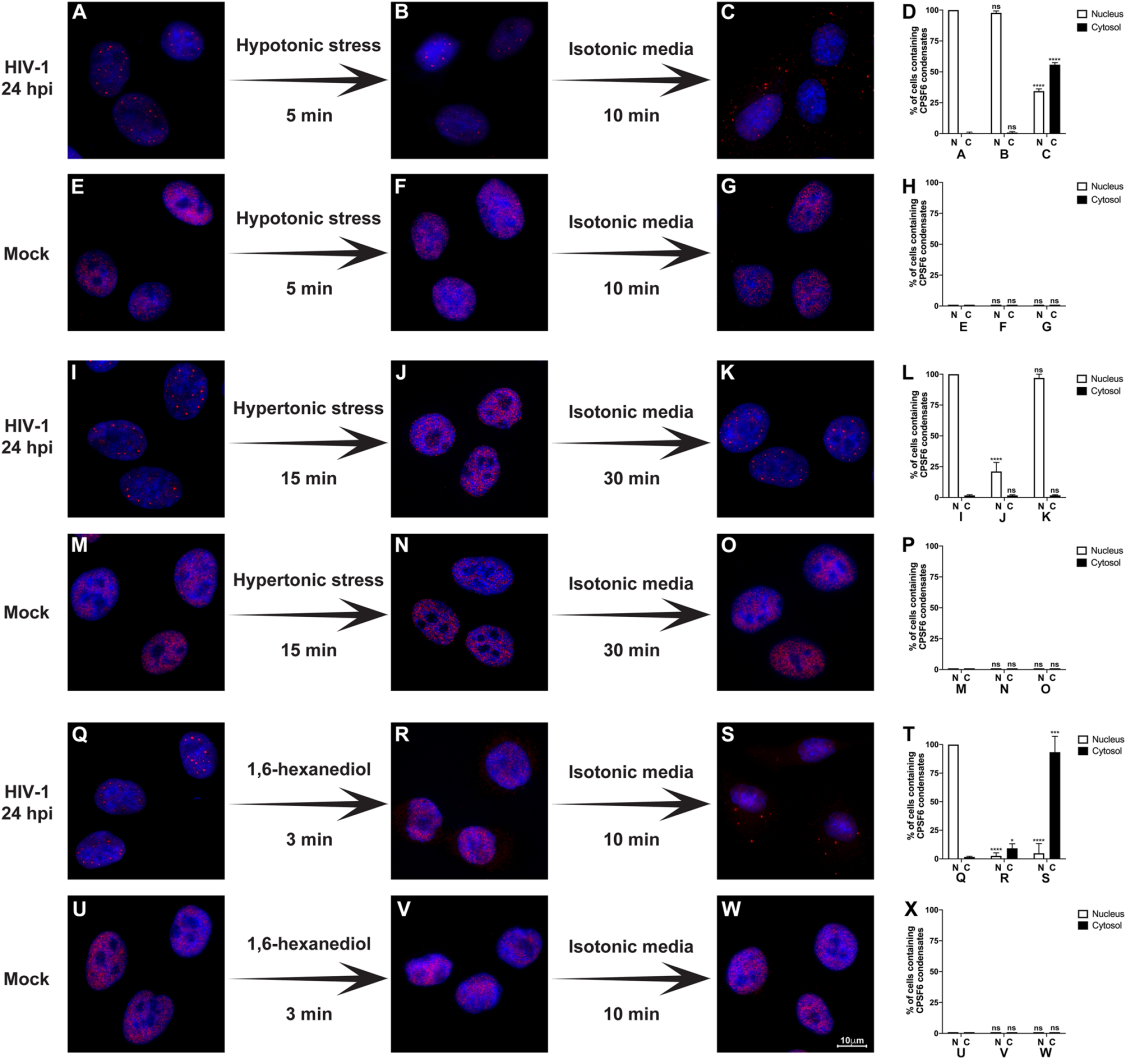
**Osmotic stress and 1,6-hexanediol affect the stability of CPSF6 condensates.** The formation of CPSF6 condensates was induced by infection of A549 cells by HIV-1-GFP at an MOI of ~ 2 for 24 h (**A**, **I**, and **Q**). Uninfected cells were used as the control (mock) (**E**, **M**, and **U**). Subsequently, infected, and uninfected cells were subjected to hypotonic stress for 5 min (**B** and **F**); hypertonic stress (200 mM NaCl-supplemented DMEM) for 15 min (**J** and **N**); or 3% 1,6-hexanediol for 3 min (**R** and **V**). After hypotonic, hypertonic, or 1,6-hexanediol treatment, the cells were incubated in isotonic medium (complete DMEM) for 10 min (**C** and **G**), 30 min (**J** and **O**), or 10 min (**S** and **W**), respectively. Cells were fixed, permeabilized, and immunolabeled with rabbit polyclonal antibody against CPSF6. The secondary antibody was Alexa-594-conjugated donkey anti-rabbit IgG. Nuclei were stained with DAPI. Stained cells were examined by fluorescence microscopy. The percentage of cells containing CPSF6 condensates in the nucleus (N) or in the cytosol (C) in the hypotonic (**D** and **H**), hypertonic (**L** and **P**), or 1,6-hexanediol (**T** and **X**) treatment was determined by visual inspection of 80 cells per sample in three independent experiments (n = 240 cells). Data represent the mean ± standard deviation. *p ≤ 0.05, **p ≤ 0.0, ***p ≤ 0.001, ****p ≤ 0.0001; ns, not statistically significant; unpaired two-tailed t test. Scale bar = 10 μm.

The formation of these CPSF6 condensates or puncta-like structures suggested that CPSF6 may be forming biomolecular condensates upon HIV-1 infection. Biomolecular condensates are non-membrane bound compartments with distinct chemical environments that are separated from their surroundings by liquid–liquid phase separation^18^. In cells, biomolecular condensates increase the local concentration of proteins to improve (1) enzymatic activity by bringing enzyme and substrate together, (2) avidity in low-affinity protein interactions, (3) nucleation for polymers such as actin and tubulin, and (4) cell signaling amplification^19^. Although cells use condensates to support their physiology, many viruses exploit biomolecular condensates for replication^20^. Because biomolecular condensates are sensitive to osmotic stress^21^, we determined whether hypotonic stress affected the stability of CPSF6 condensates. A549 cells were infected with HIV-1-GFP at an MOI of ~ 2 for 24 h to induce CPSF6 condensates (Fig. 2A). Infected cells were treated with a hypotonic medium for 5 min (Fig. 2B) and then with an isotonic medium for 10 min (Fig. 2C), which resulted in the delocalization of CPSF6 condensates from the nucleus to the cytoplasm (Fig. 2D). As a control, similar experiments were performed with uninfected cells (Fig. 2E–H). In parallel, we measured cellular viability to evaluate the effect of hypotonic treatment in cell viability by using the dye “Fixable Viability Stain 520,” which irreversibly stain dead cells. The. percentage of viable and dead cells was determined by flow cytometry. As shown in Fig. S1A, the hypotonic treatment did not affect cellular viability.

We also determined the effect of hypertonic stress on the stability of CPSF6 condensates. A549 cells were infected with HIV-1-GFP (MOI of ~ 2 for 24 h) to induce condensates (Fig. 2I) and then treated for 15 min with hypertonic medium (Fig. 2J) before they were returned to isotonic medium for 30 min (Fig. 2K). The hypertonic medium resulted in the complete disassembly of nuclear CPSF6 condensates (Fig. 2J and L); however, returning the cells to an isotonic medium allowed CPSF6 condensates to reassemble in the nucleus. Uninfected control cells showed no effects on CPSF6 localization (Fig. 2M–P). Thus, osmotic changes triggered rapid disassembly and reassembly of nuclear CPSF6 condensates, demonstrating their dynamic nature and supporting the conclusion that they are biomolecular condensates. The percentage of viable cells after the treatment was determined by flow cytometry using the “Fixable Viability Stain 520” dye. As shown in Fig. S1B, hypertonic treatment did not affect the viability of cells.

### 6-hexanediol induces the disassembly of CPSF6 biomolecular condensates

One of the hallmark features of biomolecular condensates is that they are sensitive to 1,6-hexanediol due to that this drug interferes with hydrophobic interactions and kinase activity^22–24^. CPSF6 condensates induced by HIV-1 infection of A549 cells (MOI of ~ 2 for 24 h) (Fig. 2Q) and then treated with 3% w/v 1,6-hexanediol for 3 min resulted in the disassembly of CPSF6 condensates (Fig. 2R and T). Interestingly, removal of the drug induced the reassembly of CPSF6 condensates in the cytoplasm (Fig. 2S and T). Uninfected control cells showed no effects on CPSF6 localization (Fig. 2U–X). Because it is well documented that 1,6-hexanediol dissolves and disassembles biomolecular condensates^22–24^, these experiments strengthen the conclusion that these structures are biomolecular condensates. The percentage of viable cells after the treatment was determined by flow cytometry using the “Fixable Viability Stain 520” dye. As shown in Fig. S1C, the use of 3% w/v 1,6-hexanediol for 3 min did not affect cellular viability. Next, we attempted to perform in vivo imaging experiments to visualize disassembly of the condensates utilizing CPSF6 fused to fluorescent proteins; however, transient or stable expression of CPSF6 fused to the fluorescent protein eGFP or mNeonGreen resulted in the formation of big worm-shaped aggregates in uninfected cells (Fig. S2).

### GS-CA-1 stabilizes the HIV-1-induced CPSF6 condensates in the nucleus upon hypotonic stress

**Figure 3:**
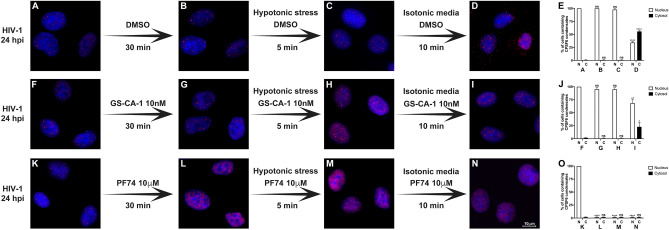
**GS-CA-1 stabilizes the HIV-1-induced CPSF6 condensates in the nucleus upon hypotonic stress in A549 cells.** To induce the formation of CPSF6 condensates, we infected A549 cells with HIV-1-GFP at an MOI of ~ 2 for 24 h (**A**, **F**, and **K**). Infected cells were incubated with DMSO (as the vehicle control) (**B**), 10 nM GS-CA-1 (**G**), or 10 μM PF74 (**L**) for 30 min. Cells were subsequently subjected to hypotonic stress with DMSO (**C**), 10 nM GS-CA-1 (**H**), or 10 μM PF74 (**M**) for 5 min. After hypotonic stress, cells were incubated in an isotonic medium with DMSO (**D**), 10 nM GS-CA-1 (**I**), or 10 μM PF74 (**N**) for 10 min. Cells were fixed, permeabilized, and immunolabeled with rabbit polyclonal antibody to human CPSF6. The secondary antibody was Alexa-594-conjugated donkey anti-rabbit IgG. Nuclei were stained with DAPI. Stained cells were examined by fluorescence microscopy. The percentage of cells containing CPSF6 condensates in the nucleus (N) or in the cytosol (C) under hypotonic stress with DMSO (**E**), 10 nM GS-CA-1 (**J**), or 10 μM PF74 (**O**) was determined as in Fig. 2. Data represent the mean ± standard deviation. *p ≤ 0.05, **p ≤ 0.0, ***p ≤ 0.001, ****p ≤ 0.0001; ns, not statistically significant; unpaired two-tailed t test. Scale bar = 10 μm.

The re-establishment of isotonic conditions after hypotonic treatment led to the relocalization of CPSF6 condensates to the cytoplasm (see Fig. 2). In addition, we previously demonstrated that the small molecule GS-CA-1 does not disrupt preformed CPSF6 condensates^11^. GS-CA-1 may stabilize CPSF6 condensates similar to the way cyclopamine stabilizes the biomolecular condensates induced by infection of respiratory syncytial virus^25^. To determine whether GS-CA-1 affects the stability of CPSF6 condensates after osmotic stress, we performed the hypotonic stress experiment using GS-CA-1 (Fig. 3A–J). As a control, we utilized the small molecule PF74, which triggers the disassembly of CPSF6 condensates^11^(Fig. 3K–O). GS-CA-1 prevented the delocalization of CPSF6 condensates to the cytoplasm (Fig. 3F–J), whereas PF74 resulted in the disassembly of CPSF6 condensates (Fig. 3K–O). Thus, GS-CA-1 stabilized CPSF6 nuclear condensates, thereby inhibiting the changes in the structure and localization of CPSF6 condensates induced by osmotic stress.

### GS-CA-1 reassembles the HIV1-induced CPSF6 condensates in the nucleus after 1,6-hexanediol treatment

**Figure 4:**
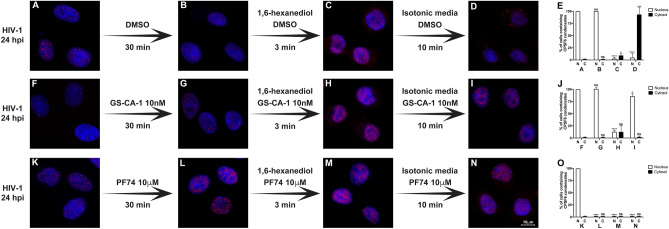
**GS-CA-1 reassembles the HIV1-induced CPSF6 condensates in the nucleus after 1,6-hexanediol treatment in A549 cells. **To induce the formation of CPSF6 condensates, we infected A549 cells with HIV-1-GFP at an MOI of ~ 2 for 24 h (**A**, **F**, and **K**). Infected cells were incubated with DMSO (**B**), 10 nM GS-CA-1 (**G**), or 10 μM PF74 (**L**) for 30 min. Cells were treated with 3% 1,6-hexanediol with DMSO (**C**), 10 nM GS-CA-1 (**H**), or 10 μM PF74 (**M**) for 3 min. After 3% 1,6-hexanediol treatment, cells were incubated in an isotonic medium with DMSO (**D**), 10 nM GS-CA-1 (**I**), or 10 μM PF74 (**N**) for 10 min. Cells were fixed, permeabilized, and immunolabeled with rabbit polyclonal antibody to CPSF6. The secondary antibody was Alexa-594-conjugated donkey anti-rabbit IgG. Nuclei were stained with DAPI. Stained cells were examined by fluorescence microscopy. The percentage of cells containing CPSF6 condensates in the nucleus (N) or in the cytosol (C) for the 1,6-hexanediol treatments with DMSO (**E**), 10 nM GS-CA-1 (**J**), or 10 μM PF74 (**O**) was determined by visual inspection of 80 cells per sample in three independent experiments (n = 240 cells). Data represent the mean ± standard deviation. *p ≤ 0.05, **p ≤ 0.0, ***p ≤ 0.001, ****p ≤ 0.0001; ns, not statistically significant; unpaired two-tailed t test. Scale bar = 10 μm.

We determined whether GS-CA-1 prevented the 1,6-hexanediol-induced disassembly of CPSF6 condensates that reassembled in the cytosol after removal of the drug (Fig. 4A–J) and found that GS-CA-1 did not prevent the disassembly of CPSF6 condensates (Fig. 4H and J) but did prevent the assembly of cytosolic CPSF6 condensates induced by the removal of 1,6-hexanediol (Fig. 4I and J). Interestingly, GS-CA-1 induced reassembly of CPSF6 condensates in the nucleus after the removal of 1,6-hexanediol (Fig. 4I and J). Control cells were treated with PF74, which induced disassembly of CPSF6 condensates (Fig. 4K–O). Thus, GS-CA-1 modulates the behavior of CPSF6 condensates. Overall, here we showed that HIV-1 induced structures containing CPSF6 exhibit biomolecular condensate behavior, which is consistent with CPSF6-GFP recovery in photobleached puncta^26^.

### Formation of CPSF6 condensates is important for wild-type HIV-1 infection

**Figure 5:**
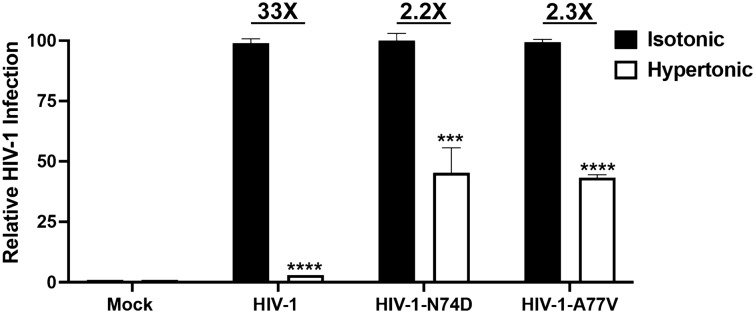
**Formation of CPSF6 condensates is required for wild-type HIV-1 infection.** A549 cells were infected wild-type or mutant HIV-1-GFP viruses at an MOI of 2, which is enough the infect more than 90% of the cells 24 h post-infection**.** At 10 h post-infection, cells were incubated or not with a hypertonic medium to prevent the formation of CPSF6 condensates. At 24 h post-infection, infectivity was measured as the percentage of GFP-positive cells using a flow cytometer. Data represent the mean ± standard deviation. *p ≤ 0.05, **p ≤ 0.0, ***p ≤ 0.001, ****p ≤ 0.0001; ns, not statistically significant; unpaired two-tailed t test.

One of the key questions in the CPSF6 area of investigation is to understand whether the induction of condensates is important for HIV-1 productive infection meaning what will happen to an HIV-1 wild type virus that cannot induce the formation of CPSF6 condensates. To understand whether the formation of condensates is important for wild type HIV-1 infection, we measured infectivity of A549 by HIV-1-GFP (MOI of ~ 2, which results in the infection of more than 90% of the cells) in cells that had been treated with a hypertonic medium at 10 h post-infection to prevent the assembly of CPSF6 condensates (Fig. 5). Remarkably, inhibiting the formation of CPSF6 condensates blocked HIV-1 infection `33 fold (Fig. 5). However, infection of mutant HIV-1 viruses bearing the capsid changes N74D or A77V, which do not induce the formation of CPSF6 condensates during infection^1,2^, were affected only ~ twofold. Wild type and mutant viruses were normalized by Western blot against p24. These experiments suggested that the formation of CPSF6 condensates is important for wild-type HIV-1 infection but not for viruses bearing the N74D or A77V capsid mutations.

### HIV-1 infection induces the formation of condensates that contain CPSF6 and CPSF5

**Figure 6:**
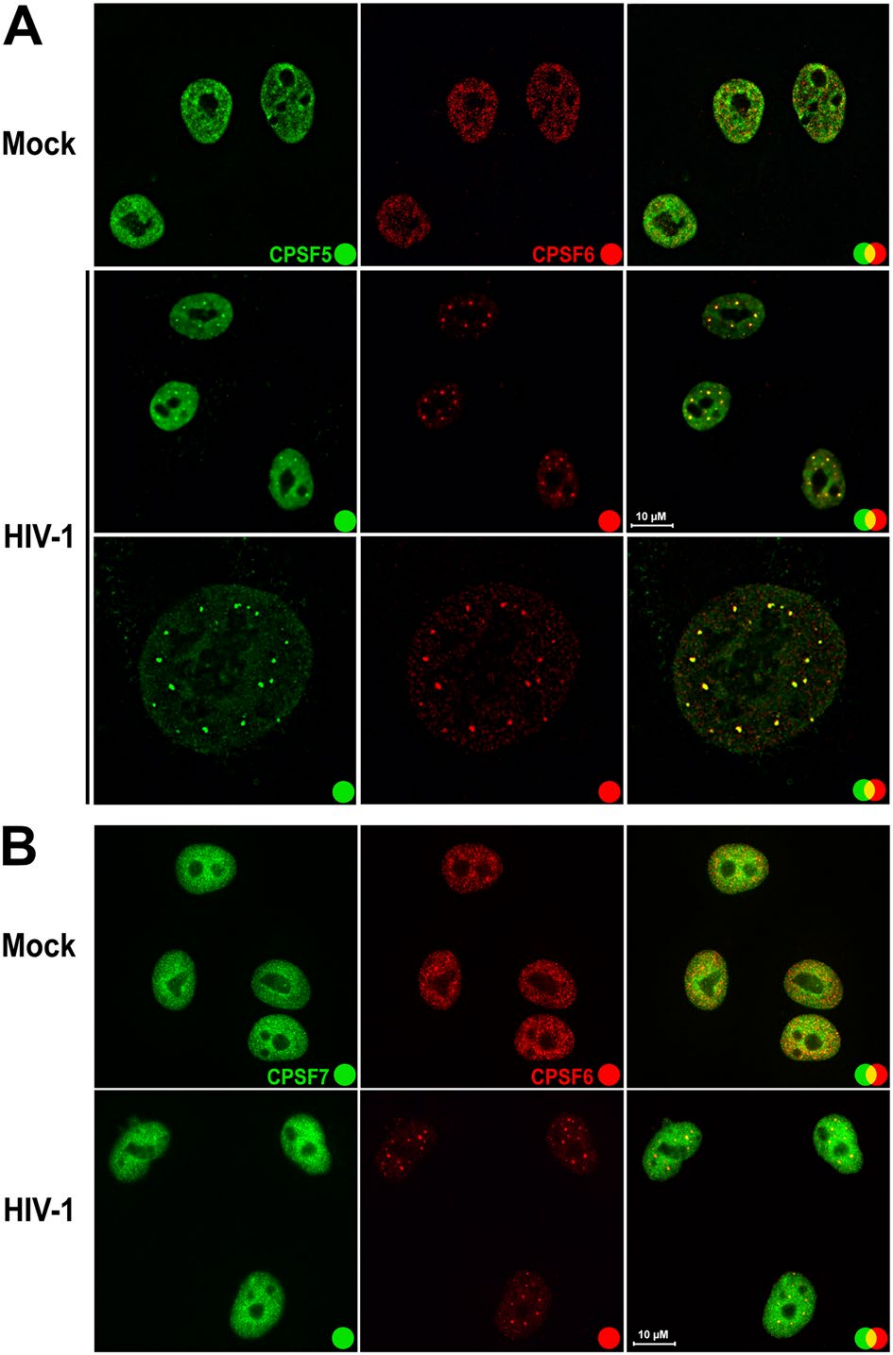
**HIV-1 infection induces the formation of CPSF5 condensates that colocalize with CPSF6.** A549 cells were infected or not (mock) with HIV-1-Luc at an MOI of ~ 2 for 24 h (**A** and **B**). Cells were fixed, permeabilized, and coimmunostained using rabbit polyclonal antibody to CPSF6 with mouse monoclonal antibody against CPSF5 (**A**) or with mouse monoclonal antibody against CPSF7 (**B**). Secondary antibodies were Alexa-488-conjugated donkey anti-mouse IgG (green channel) and Alexa-594-conjugated donkey anti-rabbit IgG (red channel). Stained cells were examined by fluorescence microscopy. Merging the red and green channels generated the third image in each row; yellow indicates overlapping localization of the red and green channels. Experiments were repeated at least three times, and a representative experiment is shown. Scale bar = 10 μm.

The cleavage factor Im (CFI_m_) is a protein complex that regulates mRNA 3′ processing and polyadenylation site selection^27^. CFI_m_ is a tetramer composed of two 25-kDa CPSF5 subunits and two proteins of either 59 or 68 kDa (CPSF7 or CPSF6, respectively)^28^. To further characterize the condensates induced by HIV-1 infection, we used colocalization experiments to assess the presence of CPSF5 and/or CPSF7 in CPSF6 condensates. CPSF6 condensates induced by HIV-1 infection colocalized with CPSF5 (Fig. 6A), indicating that these HIV-1-induced condensates contain at least two proteins, CPSF6 and CPSF5. However, we did not observe CPSF7 in HIV-1-induced condensates (Fig. 6B). These experiments indicated that HIV-1 infection induces condensates that are likely to contain the tetramer with two subunits of CPSF5 and two subunits of CPSF6. Interestingly, the CPSF5_2_-CPSF6_2_ tetramer correlates with decreased cleavage at proximal poly-A sites^29^.

2.9.1.HIV-1 induces the formation of condensates in human T cells and human primary macrophages.

**Figure 7:**
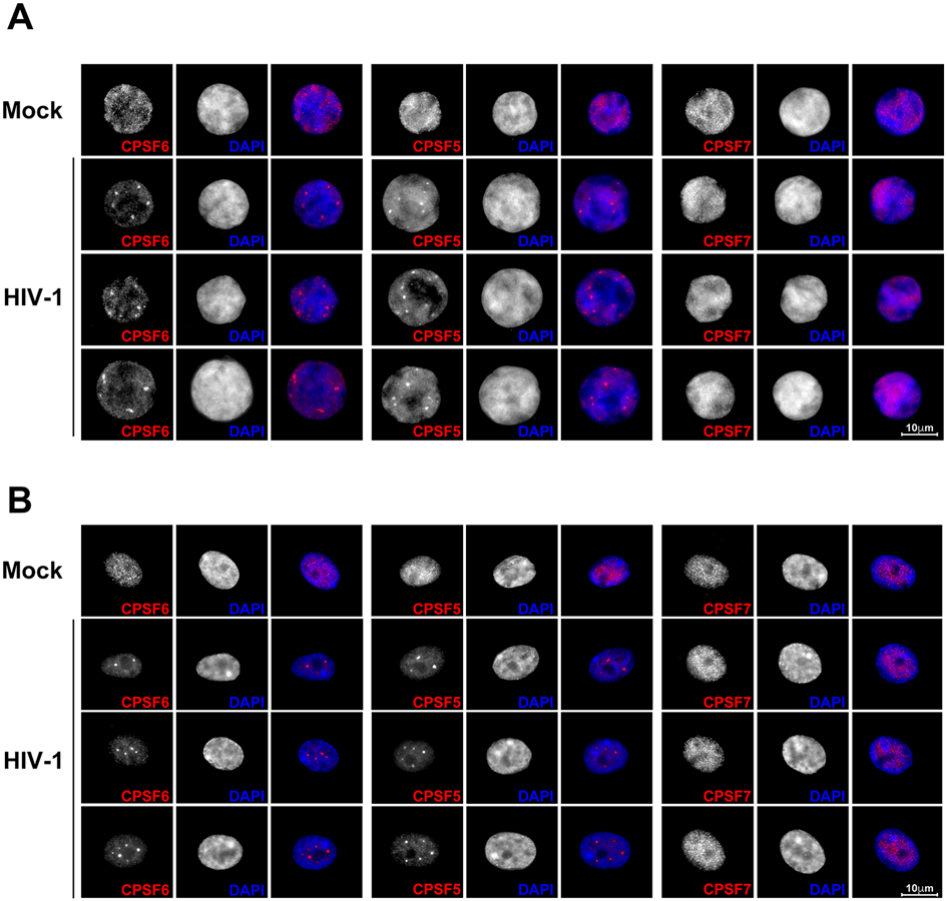
**HIV-1 infection induces the formation of CPSF6 and CPSF5 condensates in human T cells and human primary macrophages.** Jurkat T cells (**A**) and macrophages (**B**) were infected or not (mock) with HIV-1-GFP at an MOI of ~ 2 for 48 or 72 h, respectively. At 48hpi, 2,5 × 10^5^ Jurkat cells were seeded on glass coverslips previously treated with poly-D-lysine for 1 h. Cells were fixed, permeabilized, and immunostained using rabbit polyclonal antibody to CPSF6, mouse monoclonal antibody against CPSF5 or mouse monoclonal antibody against CPSF7. Secondary antibody was Alexa-594-conjugated donkey anti-rabbit IgG (red channel). Nuclei were stained with DAPI (blue channel). Stained cells were examined by fluorescence microscopy. Merging the red and blue channels generated the third image in each row; magenta indicates overlapping of the red and blue channels. Experiments were repeated at least three times, and a representative experiment is shown. Scale bar = 10 μm.

To test whether HIV-1 infection of T cells induces the formation of CPSF6/CPSF5 condensates, we challenged Jurkat T cells using HIV-1-GFP at an MOI = 2 for 48 h. The presence of condensates was evaluated by immunofluorescence using anti-CPSF6 and anti-CPSF5 antibodies in infected cells. As shown in Fig. 7A, most of the infected cells showed condensates containing CPSF6 and CPSF5. Parallel to our observations on A549 cells, human T cells did not show accumulation of CPSF7 in condensates (Fig. 7A). These results showed that HIV-1 infection of T cells, a natural target for HIV-1, induces formation of condensates containing CPSF6 and CPSF5.

Next, we tested the induction of condensates in human primary macrophages. Interestingly, HIV-1 induced the formation of large condensates in macrophages, and similar to A549 and T cells, these condensates contained CPSF6 and CPSF5 (Fig. 7B). Condensates in macrophages were also devoid of CPSF7. Overall, this section showed that condensates are formed in two different relevant targets for HIV-1, T cells and human primary macrophages.

### HIV-1 infection induces nuclear redistribution of LEDGF/p75

**Figure 8:**
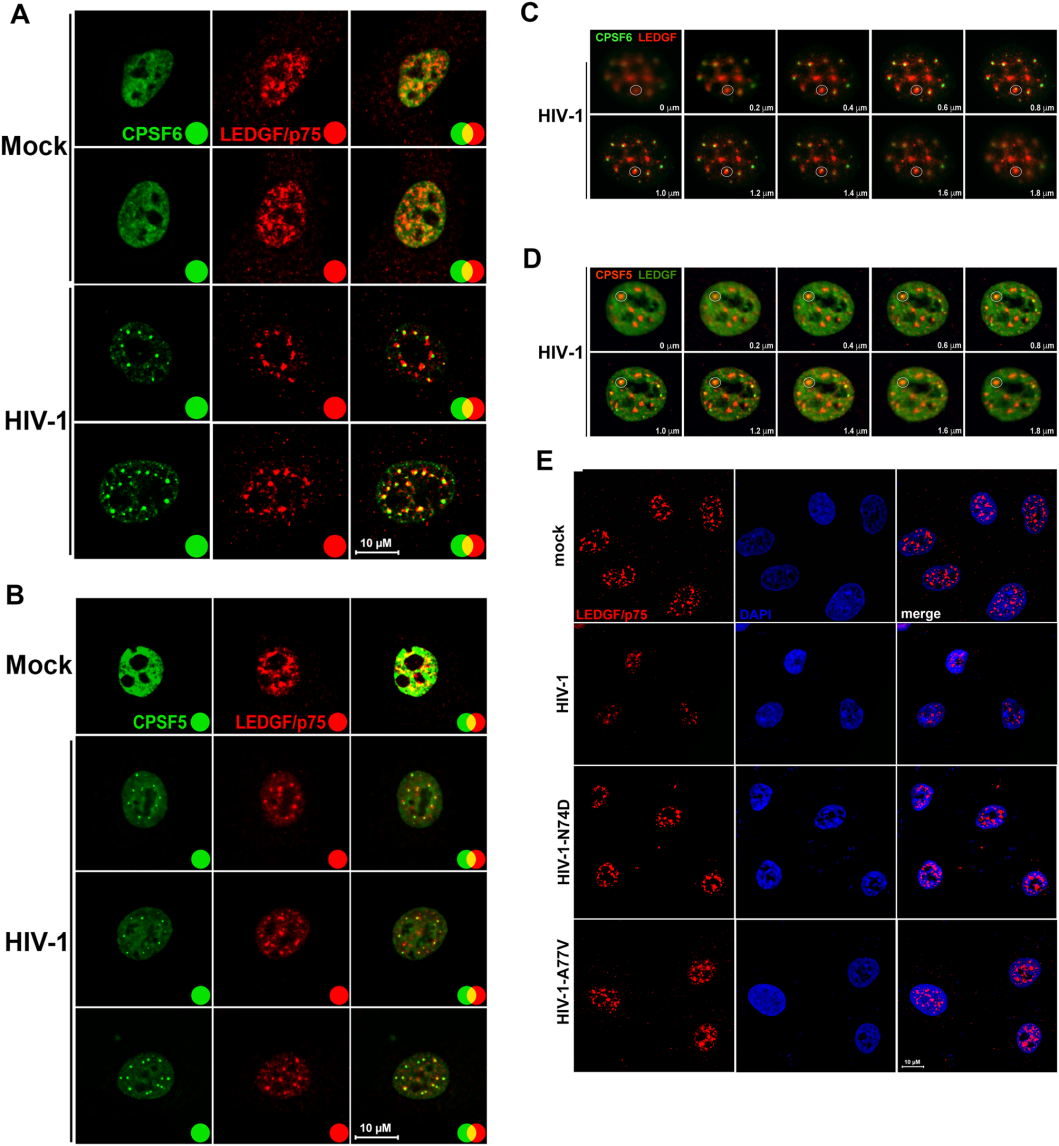
**HIV-1 infection induces nuclear redistribution of LEDGF/p75.** A549 cells were infected or not (mock) with wild-type HIV-1-Luc (**A**–**D**) or with HIV-1-GFP viruses bearing the capsid mutations N74D and A77V (**E**) at an MOI of ~ 2 for 24 h. Cells were fixed, permeabilized, and stained using rabbit polyclonal antibody to LEDGF/p75 (**E**) with mouse monoclonal antibody to CPSF6 (**A** and **C**) or with mouse monoclonal antibody to CPSF5 (**B** and **D**). Secondary antibodies were Alexa-488-conjugated donkey anti-mouse IgG (green channel) and Alexa-594-conjugated donkey anti-rabbit IgG (red channel). Nuclei were stained with DAPI (channel blue). Stained cells were examined by fluorescence microscopy. Merging the red and green channels generated the yellow color that indicates overlapping localization of these channels. Merging the red and blue channels generated the magenta color that indicates overlapping localization of these channels. For C and D z-stack images at 0.2 μm intervals were acquired. The white circle indicates an example of a condensate that contains CPSF6 (**C**) or CPSF5 (**D**) surrounded by LEDGF/p75. Scale bar = 10 μm.

LEDGF/p75 is a nuclear protein important for HIV-1 integration site selection^30^. We determined whether pLEDGF/p75 was part of the HIV-1-induced condensates. Interestingly, we observed a small amount of partial colocalization between CPSF6 and LEDGF/p75 (Fig. 8A), and the LEDGF/p75 staining surrounded CPSF6 condensates (Fig. 8A), forming a shell around these structures. To confirm these observations, we performed similar colocalization experiments using CPSF5. We observed that LEDGF/p75 also surrounded condensates that contained CPSF5 (Fig. 8B). Similarly, we also observed partial colocalization between LEDGF/p75 and CPSF5. The bi-dimensional X/Y plane shown in Fig. 8A and B suggested that condensates containing CPSF5 and CPSF6 are surrounded by LEDGF/p75 staining; however, these images do not show whether LEDFG/p75 is above or below the condensate. To answer this question, we prepared Z sections and showed that LEDGF/p75 staining is above and below the condensates containing CPSF6 and CPSF5 (Fig. 8C and D). These results demonstrated that HIV-1 infection induces the formation of a condensate that contains CPSF5/CPSF6 surrounded by LEDGF/p75. Curiously, infection by HIV-1 induced a change in the nuclear distribution pattern of LEDGF/p75 from a diffuse pattern to more puncta-like structures (Fig. 8E). However, HIV-1 viruses bearing the capsid mutation N74D or A77V, which do not induce the formation of CPSF6 condensates^1,2^, did not induce a change of LEDGF/p75 cellular localization (Fig. 8D). These experiments suggest that these HIV-1 mutants use a different pathway for infection compared to the wild-type virus.

## Discussion

We explored the role of CPSF6 condensates in HIV-1 infection. Because our previous findings suggested that the formation of CPSF6 condensates upon HIV-1 infection requires an intact capsid protein in the nuclear compartment^11^, we investigated whether the early steps of HIV-1 infection are required for the formation of CPSF6 condensates. Interestingly, genetic, or pharmacological inhibition of viral integration or reverse transcription did not affect the formation of CPSF6 condensates suggesting that neither of these processes is required for the formation of CPSF6 condensates. To our surprise, HIV-1 particles without a genome also induced CPSF6 condensates, suggesting that only the structural proteins of the virus are important for to induce formation of condensates. Finally, we found that viral particles containing only HIV-1 gag-pol and VSV-G were sufficient to induce the formation of CPSF6 condensates (data not shown). To further explore the capsid protein as a determinant for the formation of condensates, we tested mutants in other regions of the capsid, such as the cyclophilin A binding loop (capsid mutant P90A) and found this region was not important for the formation of CPSF6 condensates.

We also determined the presence of these condensates over time. We found the highest percentage of cells containing condensates at ~ 24 h post-infection, which then declined over time in the next four days, indicating that these structures may be transient. Remarkably, as the percentage of cells containing CPSF6 condensates declined, the size of the remaining condensates increased, which may indicate that fusion of condensates is occurring during cell division.

Our data showing that CPSF6 condensates were affected by osmotic stress and the drug 1,6-hexanediol indicate that HIV-1-induced CPSF6 condensates behave as biomolecular condensates. Either hypertonic stress or the drug 1,6-hexanediol triggered the rapid disassembly of CPSF6 condensates, consistent with the dynamic nature of biomolecular condensates^21,31^. Our work combined with the studies of Scoca et. al., which showed fluorescent recovery after photobleaching of CPSF6 proteins fused to neon-green fluorescent protein^26^, provide strong evidence to suggest that HIV-1-induced CPSF6 puncta are in fact biomolecular condensates, since these structures are: (1) sensitive to osmotic stress, (2) sensitive to 1,6-hexanediol, (3) growing overtime due to fusion events, and (4) recoverable upon photobleaching.

To further characterize the protein content of these condensates, we determined whether proteins that function in a complex with CPSF6 were also present in the condensates. We found that CPSF5, which forms a complex with CPSF6, was present in the condensates suggesting that HIV-1 infection induces the formation of condensates that contain the CPSF5_2_-CPSF6_2_ tetramer. However, we found no accumulation of CPSF7 in condensates suggesting that HIV-1 infection does not induce accumulation of the tetramer CPSF5_2_-CPSF7_2_.

We found that the epithelial cell line A549 is a very good model to study HIV-1 induced biomolecular condensates since condensates are formed in almost 80–85% of infected cells. However, these cells are not the natural target of HIV-1. For this reason, we investigated whether HIV-1 infection of natural targets cells, such as human T cells and primary macrophages, induce the formation of condensates. Our investigations revealed that HIV-1 infection of human T cells and macrophages induces the formation of condensates containing CPSF5 and CPSF6.

Because hypertonic stress induces the disassembly of HIV-1-induced CPSF6 condensates, we determined whether the formation of condensates is important for infection. When we prevented the formation of CPSF6 condensates using a hypertonic medium during infection, wild-type HIV-1 infection was inhibited, but HIV-1 capsid mutants N74D and A77V were much less affected by the hypertonic medium. These results are consistent with the observation that HIV-1 capsid mutants N74D and A77V do not form condensates^1,2^, and do not require CPSF6 condensates for infection. In addition, the integration pattern of HIV-1 capsid mutant N74D is very similar to the pattern shown by wild-type HIV-1 infection of CPSF6 knockout cells^12,13^. Our data showed that CPSF6 condensates are important for wild-type HIV-1 infection. It is important to point out that due to the condensing nature of some nuclear pore complex proteins, such as nucleoporins, hypertonic treatment could also have an effect on them and thus affect HIV-1 infection^32^.

Investigations to understand whether other nuclear proteins are in proximity or part of the HIV-1-induced condensates revealed that the integration cofactor LEDGF/p75 tri-dimensionally surrounds the condensate that contains CPSF6 and CPSF5. This observation suggested a potential functional link between the condensate and LEDGF/p75. Although this requires further exploration, the fact that LEDGF/p75 gets in proximity of the condensate forming a surrounding layer raises the possibility of a second condensate or environment that will facilitate infection.

## Materials and methods

### Cell culture

A549 (human lung carcinoma), HT1080 (human fibrosarcoma), and HeLa (human epithelium) cells were obtained from the American Type Culture Collection (ATCC; Cat# CCL-1885, Cat# CC-121, and Cat# CCL-2), and were maintained in Dulbecco's Modified Eagle Medium (DMEM) supplemented with 10% heat-inactivated fetal bovine serum (FBS), 100 U/ml penicillin, 100 μg/ml streptomycin, 29.2 mg/ml L-glutamine (Life Sciences), and 5 μg/ml plasmocin (Invivo Gen, San Diego, CA), in a humidified incubator with 5% CO_2_ at 37 °C. Human THP-1 and U937 monocytes and human Jurkat T cells were obtained from ATCC (Cat# TIB-202, CRL-1593.2 and Cat#TIB-152) and maintained in Roswell Park Memorial Institute medium (RPMI) supplemented with 10% FBS and 1% penicillin/ streptomycin/ L-glutamine in a humidified incubator with 5% CO_2_ at 37 °C. Monocytes were isolated from peripheral blood mononuclear cells (PBMCs) from healthy donor using the Pan Monocytes Isolation kit from Miltenyi Biotec (130-096-537). Monocytes were differentiated into macrophages by culturing in DMEM supplemented with 10% human serum for 7 days. The A549 cell lines that stably express the restriction factors TRIM5α_rh_ or TRIMCyp were previously generated (Selyutina et al., 2020). HeLa cell line stably expressing CPSF6 fused at C-terminal to the enhanced green fluorescent protein (eGFP) was generated by retroviral transduction.

### Antibodies and cell reagents

We used the following mouse monoclonal antibodies: clone SC-35 against SC35 (Cat# ab11826, Abcam), clone 3F8 against CPSF5 (Cat# H00011051-M12, Novus Biologicals), clone A-9 against CPSF7 (Cat# sc-393880, Santa Cruz) and clone F3 against CPSF6 (Cat# sc-376228, Santa Cruz). We used rabbit polyclonal antibodies against the following proteins: CPSF6 (Cat# ab99347, Abcam) and LEDGF/p75 (Cat# A300-847A, Bethyl Laboratories, Inc). The fluorescent nuclear stain 4’,6-diamidino-2-phenylindole (DAPI) and the following fluorescently labeled antibodies were from Life Technologies: Alexa Fluor 488-conjugated and Alexa Fluor 594-conjugated donkey anti-rabbit IgG and anti-mouse IgG. The inhibitors nevirapine (Nev) (Cat# ARP-4666), zidovudine (AZT) (Cat# ARP-3485), and raltegravir (Ral) (Cat# ARP-11680) were from NIH AIDS Reagent Program. Dimethyl sulfoxide (DMSO) (Cat# D2438), cyclosporin A (CsA) (Cat# 30024), and PF74 (Cat#SML0835) were from Sigma Aldrich. GS-CA-1 was kindly provided by Stephen Yant (Gilead Sciences, CA).

### Production of HIV-1 viruses

Wild-type and mutant HIV-1 (N74D, D185N, D116N, P90A, A92E, A77V, N57S, G208R, or A14C/E45C) expressing green fluorescent protein (GFP) or luciferase (Luc) as a reporter gene were produced by cotransfecting HIV-1-gag-pol, LTR-GFP-LTR/LTR-Luc-LTR, tat, rev and VSV-G in HEK293T/17 cells as described^33^. Viruses were collected 48 h post-transfection, filtered, titered, aliquoted, and stored at − 80 °C.

### Analysis of viral infectivity by flow cytometry

The infectivity and titer of HIV-1-Luc viruses were measured using TZM-bl GFP-reporter cells in which HIV-1 infection induces GFP as described^34^. We infected human A549 cells and determined the percentage of GFP-positive cells using a flow cytometer (BD Celesta). For mutant viruses that affect the infectivity of HIV-1, we normalized the amount of virus against p24 by ELISA and/or western blotting using antibodies against p24 (NIH AIDS repository). Virus titer calculation was performed according to the following equation: Infectious units (IU)/ml = (cell number) × (% of GFP-positive cells) × (dilution factor), where the dilution factor = 1000 μl/viral input (μl). To calculate the volume of virus used at a specific MOI, use the following equation: MOI = [(virus stock IU/ml) × (volume of virus used)]/(number of cells in infection).

### Osmotic stress and 1,6-hexanediol treatments

For osmotic stress treatments, the isotonic medium (~ 300 mOsm/H_2_O) was DMEM supplemented with 10% FBS and 1% penicillin/streptomycin/L-glutamine. For hypotonic stress (30 mOsm/H_2_O), tenfold diluted DMEM (supplemented with 10% FBS and 1% penicillin/streptomycin/L-glutamine) with ultrapure H_2_O was used^25^. For hypertonic stress (~ 500 mOsm/H_2_O), DMEM was supplemented with 10% FBS, 1% penicillin/streptomycin/L-glutamine and 200 mM NaCl. For 1,6-hexanediol treatment, DMEM was supplemented with 10% FBS, 1% penicillin/streptomycin/L-glutamine and 3% 1,6-hexanediol.

### Immunofluorescence microscopy and image acquisition, and deconvolution

Samples for immunofluorescence analysis were prepared as described previously with some modifications^2^. Briefly, cells were seeded on 12-mm round, glass coverslips in a 24-well plate and maintained in a complete culture medium. After HIV1 infection and/or drug or osmotic treatments, the coverslips were rinsed with PBS and fixed with 4% paraformaldehyde-PBS for 15 − 30 min at room temperature. Subsequently, cells were incubated in 0.1 M glycine-PBS for 10 min at room temperature. Cells were permeabilized using 0.5% Triton X-100 for 5 min at room temperature. Non-specific binding was prevented using blocking solution, 3% bovine serum albumin in PBS, for 60 min at room temperature. Samples were incubated with primary antibodies in blocking solution for 60 min in a dark room at room temperature. Subsequently, the coverslips were rinsed with PBS and incubated with the appropriate secondary antibodies against mouse or rabbit IgG in blocking solution for 30 min at room temperature. Finally, the coverslips were washed with PBS and mounted using FluorSave reagent (Sigma Aldrich). Fluorescence microscopy images were acquired with AxioObserver.Z1 microscope equipped with a PlanApo 63× oil immersion objective (NA 1.4) and an AxioCam MRm digital camera (Carl Zeiss). Image acquisition was carried out using a Zeiss Z1 Observer inverted microscope using the ZEN 3.3 (blue edition) software. Image deconvolution was performed with the ZEN 3.3 software using an acquired point spread function. Images for figures were processed with Adobe Photoshop CS5 software (Adobe Systems, Mountain View, CA).

### Quantification and statistical analysis

The mean and standard deviation values were calculated using GraphPad Prism 8. Statistical analysis was performed using unpaired t tests.

## Supplementary Information

Supplementary Figure S1.

Supplementary Figure S2.

## Data Availability

The datasets used and/or analyzed during the current study available from the corresponding author on reasonable request.
