# Supplementary material for: Formation of nuclear CPSF6/CPSF5 biomolecular condensates upon HIV-1 entry into the nucleus is important for productive infection
Source: Sci Rep. 2023 Jul 6;13:10974. doi: 10.1038/s41598-023-37364-x (PMC10325960; doi:10.1038/s41598-023-37364-x)
Supplement: Supplementary file 1 — Supplementary Figure S1. [file 41598_2023_37364_MOESM1_ESM.docx]

**
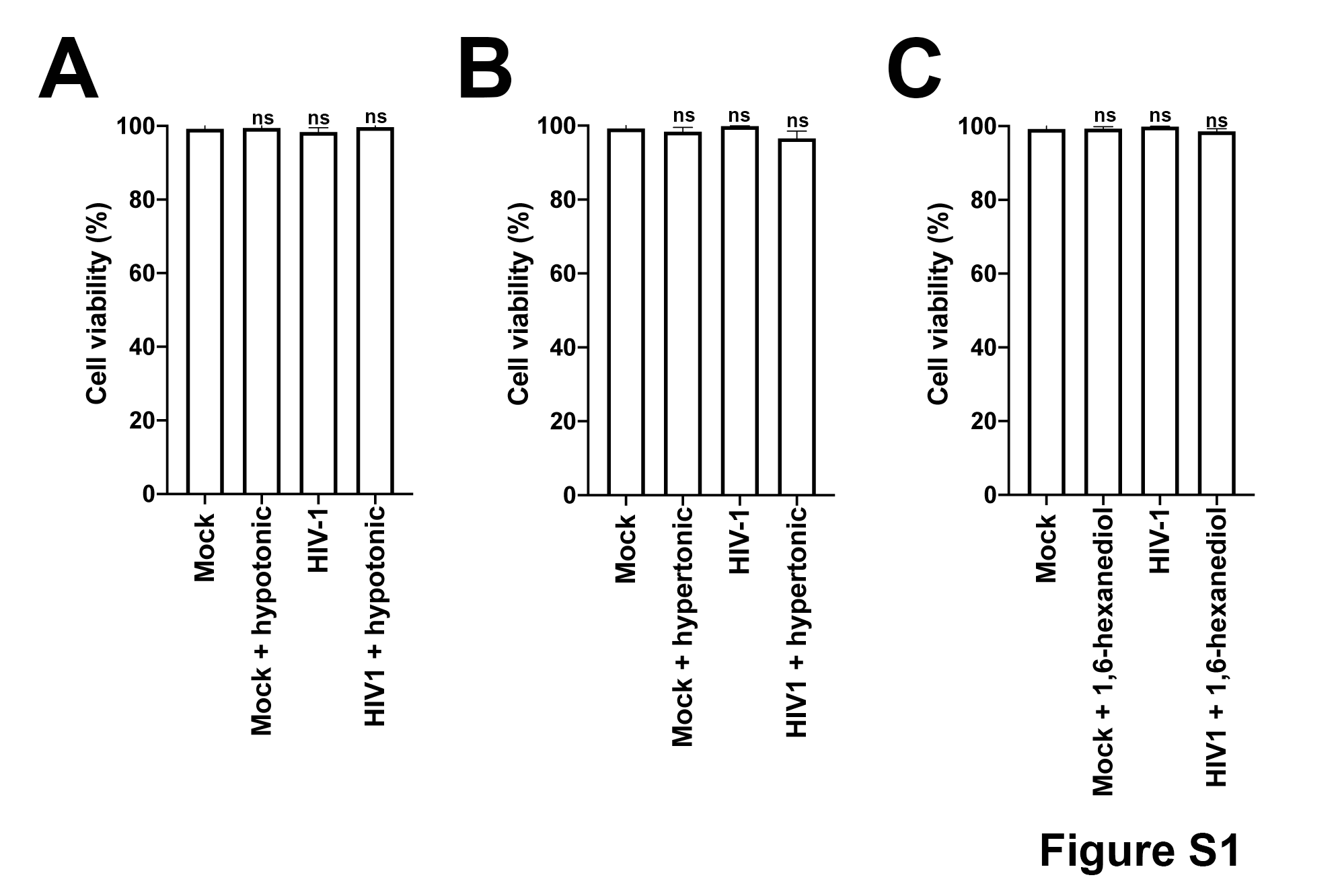
**

**Figure S1. Osmotic stress and 1,6-hexanediol do not affects the viability in A549 cells.** Formation of CPSF6 condensates was induced by infection of A549 cells by HIV-1-LUC at an MOI of ~2 for 24h **(A-C).** Uninfected cells were used as control (Mock) **(A-C).** Subsequently, infected and uninfected cells were subjected to hypotonic stress for 5 min **(A)**; hypertonic stress (200nM NaCl-supplemented DMEM medium) for 15min **(B)**; or 3% 1,6-hexanediol for 3min **(C)**. After hypotonic, hypertonic or 1,6-hexanediol treatment, cells were stained with BD Horizon^TM^ Fixable Viability Stain 520 according to the manufacturer's indications. The percentage of viable A549 cells was determined using a flow cytometer. Data represents the mean ± standard deviation of three experiments. *P≤ 0.05; **P≤ 0.01; ***P≤ 0.001; ****P≤ 0.0001; ns, not statistically significant; Unpaired t test with two-tailed.
