# Supplementary material for: Formation of nuclear CPSF6/CPSF5 biomolecular condensates upon HIV-1 entry into the nucleus is important for productive infection
Source: Sci Rep. 2023 Jul 6;13:10974. doi: 10.1038/s41598-023-37364-x (PMC10325960; doi:10.1038/s41598-023-37364-x)
Supplement: Supplementary file 2 — Supplementary Figure S2. [file 41598_2023_37364_MOESM2_ESM.docx]

**
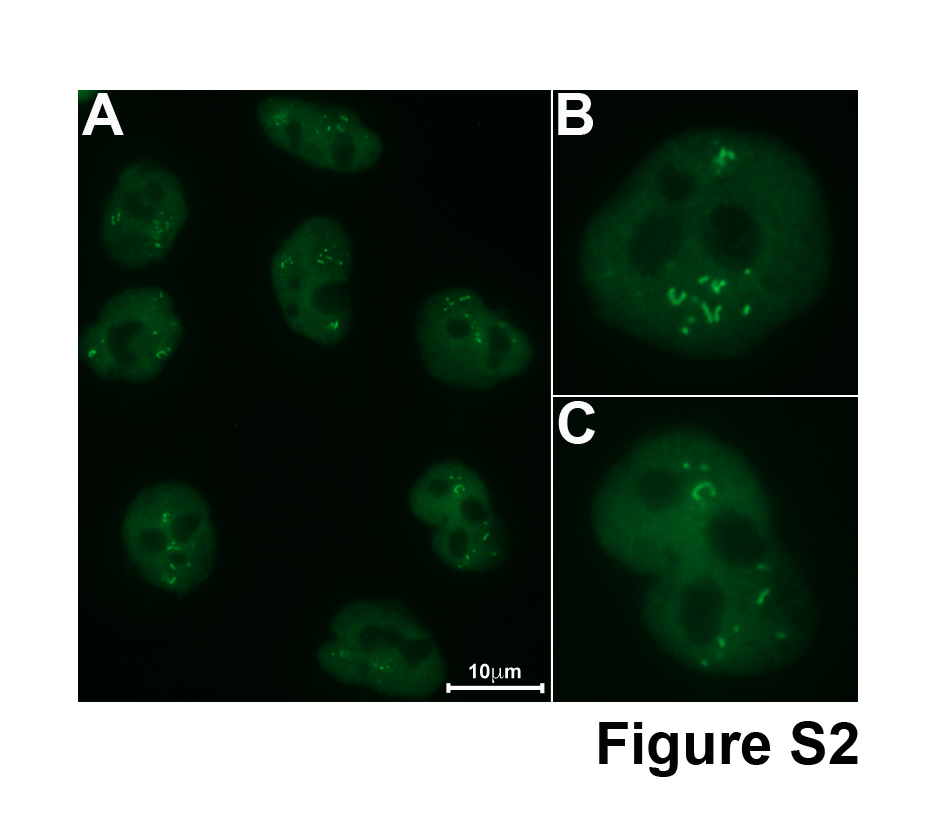
**

**Figure S2. Expression of CPSF6-eGFP induced the formation of protein aggregates.** HeLa cells stably expressing CPSF6 fused at C-terminal to enhanced green fluorescent protein (eGFP) were seeded on glass coverslips for 24h **(A-C)**. Subsequently, cells were fixed and mounted on glass slides. Cells were examined by fluorescence microscopy. **B** and **C** correspond to the zoom of a single cell in panel **A**.
